# Why are girls still dying unnecessarily? The need to address gender inequity in child health in the post–2015 development agenda

**DOI:** 10.7189/jogh.05.020303

**Published:** 2015-12

**Authors:** John Jungpa Park, Luciana Brondi

**Affiliations:** 1 Royal Free Hospital, Royal Free London NHS Foundation Trust, National Health Service, London, UK; 2 Centre for Population Health Sciences, University of Edinburgh Medical School, Edinburgh, Scotland, UK

The 40–year anniversary of the United Nations ‘International Women’s Day,’ was celebrated on 8 March 2015. As we approach the end of the Millennium Development Goals (MDGs), we reflect on the gender debate that has arose amidst tackling MDG4 and highlight the need for greater gender equality in measuring child health outcomes in the post–MDG era in line with MDG 3 (see **Box 1**).

Box 1 **Summary of Millennium Development Goals 3 and 4**

Goal 3: Promote gender equality and empower women

Eliminate gender disparity in primary and secondary education, preferably by 2005, and in all levels of education no later than 2015

3.1 Ratios of girls to boys in primary, secondary and tertiary education

3.2 Share of women in wage employment in the non–agricultural sector

3.3 Proportion of seats held by women in national parliament

Goal 4: Reduce child mortality rates

Reduce by two–thirds, between 1990 and 2015, the under–five mortality rate

4.1 Under–five mortality rate

4.2 Infant mortality rate

4.3 Proportion of 1 year–old children immunised against measles

NEED TO PROFILE GENDER AS A DETERMINANT OF CHILD HEALTH INEQUITY

In recent years, several key UN reports and articles have begun to articulate the gender gap that exists in child health outcomes [1–3]. Indeed, it has been the UN which has taken a lead in promoting gender equality internationally by requiring all UN entities to mainstream gender and promote gender equality as mandated by the Beijing Platform for Action (1995) and ECOSOC resolutions 1996, 1997, 2006 and consolidated by the quadrennial comprehensive policy review 2012 (General Assembly Resolution 67/226). According to the 2012 World Development Report, gender equality is at the heart of development and “…too many girls and women are still dying in childhood and in the reproductive ages” [4]. Perhaps it is a reflection on the relative success of MDG 3 and 4 (despite it not being likely that the numerical targets will be achieved in time) that it has helped to raise the issue of gender in child health and the need for more equitable goals in the future.

Leading international organisations have developed organisation specific gender action plans, policies or guidelines in the past two decades in order to tackle gender imbalance issues in its organisational activities (see **Box 2**).

Box 2 **Organisations identified through a Google Scholar search of ‘gender’ or ‘sex’ and ‘policy’ or ‘guideline’ or ‘framework’**

African Development Bank

Asian Development Bank

Bill and Melinda Gates Foundation

Council of Europe

Department for International Development (DFID)

European Union

Global Alliance for Vaccines and Immunisation (GAVI)

Global Fund

The International Federation of Red Cross and Red Crescent Societies (IFRC)

Organisation for Economic Co–operation and Development (OECD)

Save the Children

The United Nations Children's Fund (UNICEF)

World Bank

World Health Organisation (WHO)

The authors congratulate recent efforts to collect gender disaggregated child health outcomes data by Inter-Agency Group for Child Mortality Estimation (IGME) and Countdown 2015 as the first step to enable the profiling of gender as a determinant of child health inequity. Nevertheless, if gender is to be mainstreamed as a determinant of child health, future country achievement profiles should require nations to highlight sex disparities in coverage of life saving interventions, especially in countries where girls are known to be subject to discrimination in health care access and outcomes. In other words, it should become the norm, rather than the exception, to report sex–differentiated data for child health indicators. In addition, reporting health interventions which have been proven to reduce maternal, newborn and child mortality rates by gender would prove valuable to better realign services and make targeted policy steps.

In response to the challenge of collecting better gender data and developing an effective response, we discuss some of the challenges reported in the literature of researching gender and child health and their potential solutions. We also look briefly at the example of India; one country in which there is evidence of severe discrimination against girls in child health care outcomes, to provide a perspective of the challenge that remains ahead.

## RESEARCH ISSUES AND SOLUTIONS: GENDER AND CHILD HEALTH

### Data recording

There are major challenges to determine whether improvements in child survival are seen in both males and females. The UN Sex Differentials in Childhood Mortality [5] suggests that “[t]his is due to the inadequate nature of birth and death statistics in most developing countries. In the absence of complete vital registration, mortality estimates for these countries are derived primarily from sample surveys and population censuses, through questions posed to women about the survival of their children. Such estimates can be subject to a great deal of uncertainty due to small sample sizes, as well as biases affecting the consistent reporting of all children.”

The problems of data recording and collection have been further complicated by use of different surveys over different time periods and non–systematic methodologies, making comparisons challenging.

In order to address this problem, IGME was formed in 1994 to provide a uniform source of estimation for child mortality, and has produced sex–disaggregated data since the publication of UN’s Sex Differentials in Childhood Mortality in 2011. This marks a significant advance towards profiling and subsequently tackling the issue of gender inequities in child health and mortality.

However, there is a need for more and better quality evidence on the role of gender in child health achievements both globally and regionally. Identifying and incorporating indicators beyond generic health and disease outcomes by sex is crucial to understand how to modify the impact of gender based discrimination. Disaggregated data that incorporates age, region within a country, wealth and education of the family are important covariates to be studied in relation to gender when looking at child health care access and outcomes. Fostering research in gender inequality in child health is essential to allow for a more detailed analysis to characterise the precise scale and nature of the inequity and to make a substantial stab at the problem.

### Biological sex differences

There is evidence in the literature to suggest that females have a biological advantage in survival over males up to age 5 years, but especially in the 1st year of life, due to being less vulnerable to congenital disease, infection, and perinatal illness including perinatal trauma, intrauterine hypoxia, birth asphyxia, prematurity, neonatal tetanus and acute respiratory distress syndrome [6]. The survival advantage for girls tends to increase as total mortality levels for a country decrease and this is postulated to be associated with distributions in the causes of death [7]. In developed countries, infectious diseases account for a lower number of causes of death and perinatal, congenital and external causes form a larger proportion of deaths between ages 1–5. Therefore, the female advantage in child mortality would increase assuming that there is no health discrimination based on sex [8,9].

**Figure:**
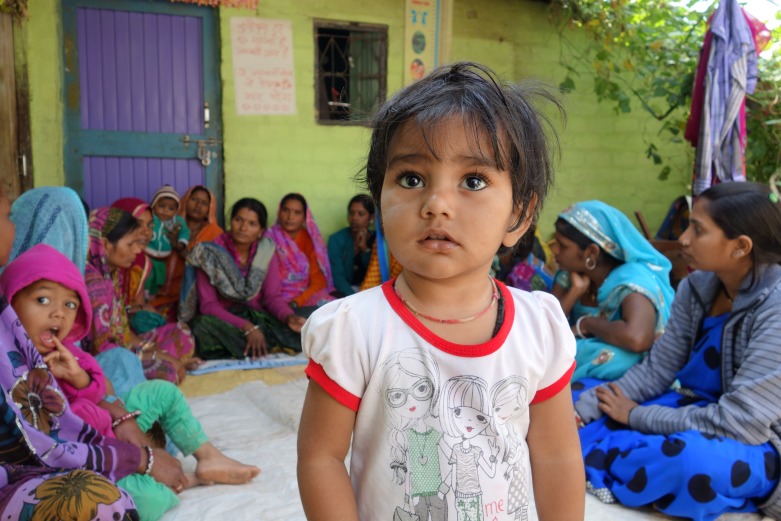
Photo: Courtesy of Indrani Kashyap, personal collection

With these expected biological advantages taken into consideration, can we profile which countries have the worst records for gender inequity for under–five (U5) mortality?

### Post–Alkema: Using estimated–expected mortality ratios

We have profiled the excess U5 mortality using data from Alkema et al., which has updated Sawyer’s model [2], to look at excess female mortality using a novel method of estimated–to–expected mortality ratios [3]. Using a Bayesian hierarchical time series approach, Alkema et al. estimate country‐specific mortality sex ratios for infants and U5 children for 195 countries from 1990 to 2012. They simultaneously assess the relationship of these mortality estimates with population sex ratios to highlight the expected and the excess female mortality rates in countries with outlying sex ratios. The authors identified 15 countries with outlying U5 sex ratios, and among these, 10 had higher than expected female mortality in 2012. For the majority of these countries the excess female mortality decreased since 1990; however, the estimated–to–expected female mortality did not change substantially for most countries except in India, where they worsened. **Table 1** shows the 10 countries that had higher than expected U5 female mortality; namely, Afghanistan, Bahrain, Bangladesh, China, Egypt, India, Iran, Jordan, Nepal, and Pakistan. We included in this table the ratio of estimated–to–expected female mortality rate, the number of excess female mortality for U5s and ratio of excess female deaths to total number of deaths (%). Countries are ranked in order of highest number of excess deaths (**Table 1**). India appears as the top country in terms of excess female U5 deaths.

**Table 1:** Indicators for 10 countries with higher than expected excess U5 female mortality and outlying under–five (U5) sex ratios in 2012*

Country	Ratio of estimated–to–expected U5 female mortality rate	Number of excess female deaths	Ratio of excess U5 female deaths to total number of deaths (%)
India	1.30 (1.26–1.34)	166 000 (144 000–190 000)	11.7
Pakistan	1.06 (1.01–1.12)	11 100 (1000–21 400)	2.7
China	1.08 (1.02–1.16)	8690 (2330–16 100)	3.3
Bangladesh	1.06 (1.01–1.11)	3330(790–5880)	2.6
Afghanistan	1.06 (1.01–1.11)	2810 (330–5390)	2.7
Egypt	1.13 (1.11–1.16)	2250 (1860–2660)	5.6
Iran	1.13 (1.06–1.20)	1340 (590–2190)	5.2
Nepal	1.08 (1.02–1.15)	852 (227–1520)	3.5
Jordan	1.12 (1.04–1.21)	188 (63–333)	5.0
Bahrain	1.14 (1.07–1.22)	11 (6–18)	5.9

*Adapted from Alkema et al. [3]. U5 mortality is defined as the probability of dying between birth and the exact age of 5 y. Sex ratio is defined as number of males per 100 females in the population, usually normalized to 100.

Clearly, however, as Alkema et al. state [3], the monitoring of sex differences in U5 mortality is complicated by variability in data availability, quality (usage and often non–usage of standard errors or uncertainty intervals), changes in country specific sex differentials over time, and validation of estimates. These findings reinforce our original point for the need of better and standardised data for all countries when it comes to gender inequality analysis in child health estimates.

## ISSUES IN INDIA: A BRIEF OVERVIEW

**Figure 1:**
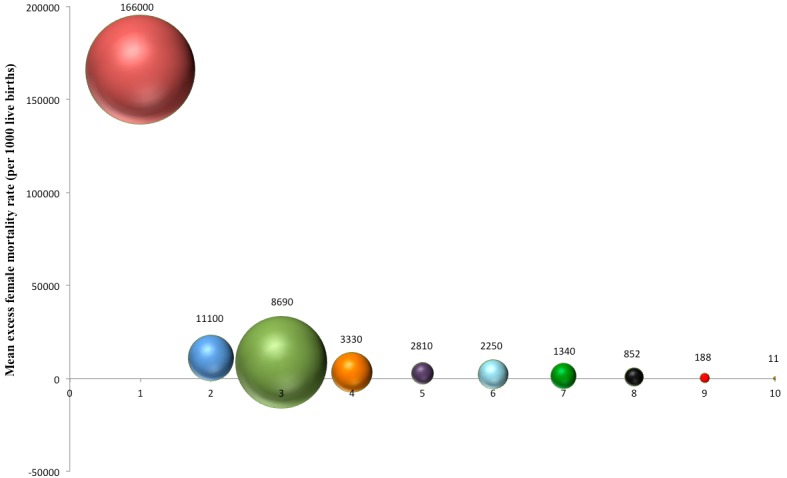
Ten countries with higher than expected excess under–five (U5) female mortality and outlying U5 sex ratios in 2012. Legend: 1 – India, 2 – Pakistan, 3 – China, 4 – Bangladesh, 5 – Afghanistan, 6 – Egypt, 7 – Iran, 8 – Nepal, 9 – Jordan, 10 – Bahrain. The bubble chart was created using UNICEF statistics and data from Alkema et al. [3] to demonstrate the 10 countries with outlying U5 sex ratios and higher than expected excess female U5 mortality. Countries are ranked in order of highest ratio of excess female U5 mortality to total number of U5 mortality. The size of the bubble corresponds to the total U5 population in each country, emphasizing the importance of addressing gender issues in child health in countries with large child populations. Source: UNICEF statistics, available at http://data.unicef.org/resources.

Globally, India has the largest number of child deaths and possesses significant regional variations in U5 mortality [10]. It accounts for the largest burden of excess female deaths than any other country in the world (**Figure 1**). The 2011 Indian census estimated that there were approximately 7.1 million fewer females than males aged 0–6 years, which was an increase from 6 million recorded in the 2001 census and 4.2 million in the 1991 census [11]. In fact, females between 1–59 months in every region in India had higher mortality compared to males [12]. Ram et al. showed significant regional variations in U5 mortality and through detailed analysis showed that the nine poorest states contained half of all people in India and just over half of all births but 71% (1 million of the 1.5 million) of deaths in children U5, highlighting the added level of regional complexity to existing gender disparities which needs to be considered for a national strategy [10].

The biggest contributor to gender imbalance in children aged 0–6 in India is likely to be prenatal sex determination with subsequent abortion of female fetuses; a practice which has increased substantially in the past 2 decades [11]. Nevertheless, there is extensive literature which also demonstrates a clear female disadvantage in health care provision and disease outcomes. For example, female children are less likely to be immunized, receive medical attention, receive appropriate antibiotic therapy or achieve good nutrition [13–15]. Therefore, to tackle gender discrimination in child health, a two-pronged approach is critical to success, addressing sex determination pre-birth and tackling discrimination in health access, preventive health and nutrition after birth.

Das Gupta et al. have argued that disparities in child health outcomes are mainly a result of a society which values its sons far over and above, and at the cost of its daughters [16]. This is a phenomenon deeply rooted in cultural, legal, social and historical reasons; hence there is a critical need for cross–disciplinary studies to help explain the gender disparities in India and guide the development of gender sensitive solutions within health care and beyond.

The government has an important role to play. Previous policies have failed to be fully effectual, and efforts to ban the sex selective abortion of females has been limited by limited by poor implementation at the state and local level [17–19]. More recently, the Government has shifted the focus to small administrative areas through the National Rural Health Mission launched in 2005 [20] and more recently the National Urban Health Mission [21]. Ram et al. have estimated that at current rates of progress MDG4 will be achieved by India in 2020, by richer states in 2014, and by poorer states in 2023 [10]. Clearly, there is a still long way to go. More efforts are needed to ensure that greater gender equality is achieved in reaching these targets across all regions in India; work that incorporates better data and research, more collaboration across sectors and agencies, and strong and effectual government policies that are based on evidence.

## CHALLENGES AND RECOMMENDATIONS FOR FUTURE WORK

There is a wide scope for future work into gender and child health. It is not only an important area of research, but also at present, an under–appreciated one. In particular, we have highlighted the need for progress in India, which has the largest number of excess female mortality and is home to one fifth of all children in the world (**Figure 1**).

The need for better quality data and research in child health and gender is unquestionable [22]. The global scientific community has a central role to play in the efforts to unmask, characterise, and explain the issues in a language that makes sense to governments and the international community; this is at the core of helping governments and international organisations to implement evidence based policies and programmes. Indeed, if gender is to be mainstreamed as a determinant of child health, future country achievement profiles should require all nations to highlight sex disparities in mortality and coverage of life saving interventions. As the evidence in India highlights, there are two key time points in gender bias; pre–birth and post–birth. More studies are needed to look at both prenatal sex determination and health access and outcomes in children.

Gender is commonly thought to be a development problem and therefore, tackling development issues such as poverty and education, could be seen as a good response to gender discrimination in child health care. However, studies in India have demonstrated that gender based discrimination against women has deep social and cultural roots and relates to family organisation norms [23,24]. There is evidence that gender bias against girls has become so deeply–rooted in some South Asian countries, and that it persists or worsens in more educated and richer families, compared to those who are poorer and less educated [25]. In the last two decades, both biomedical and social researchers have collected and analyzed evidence on different aspects of sex differentials in mortality especially in children. However, there is still a need for a more comprehensive model explaining these differentials and including the biological, social, cultural and economic factors. Further research, which incorporates the determinants of health, could help tackle discrimination against girls in different contexts [22].

Gender inequity in child health is certainly an important global health issue that requires a global solution. Addressing gender bias in child health formally in a post–2015 development agenda would give greater impetus for a more effectual and coordinated global effort to invest, address, and make progress to reduce the inequity. Indeed, when future improvements in health outcomes for children are made globally in the post–MDG era, they should be recognized for being equitable as they are now for reaching total targets.
